# The economic effect of applying Ayurveda in countries health care

**DOI:** 10.1186/1878-5085-5-S1-A102

**Published:** 2014-02-11

**Authors:** Anita Karilio-Arkas

**Affiliations:** 1 Ayurveda Russia-India Association, Moscow, Russia

## 

In opinion of experts of World Health Organization, “the global burden and threat of non-infection diseases are the greatest threat to public health and undermine social and economic development in the whole of the world”. (WHO’s global strategy in prevention of non-infection diseases and control thereof.)

It is estimated that 36 lethal cases, or 63% of 57 million of all lethal cases recorded in the world in 2008, were caused by non-infection diseases, including, first of all, cardiovascular diseases (48% cases of non-infection diseases), oncology diseases (21%), chronic respiratory diseases (12%) and diabetes (3.5%).

According to WHO’s prognosis, the total number of lethal cases caused by non-infection diseases will reach 55 million per year by 2030, unless proper measures are taken.

In the modern allopathic medicine the preventive care lays in the layer of early diagnostics, i.e. at the moment when a patient is ill already. Significance of preventive care is hard to be overestimated; budgetary funds assigned for prevention of disease are much smaller than those for its treatment. Priority of preventive care is fixed as a basic principle; in this relation, integration of 4P medicine in health care, transition of physicians and health care managers from predominantly treatment and diagnostic to predominantly predictive, preventive, prophylactic, personalized processes at patients’ active participation will lead to elevation of life quality of the population.

The current situation, as specialists of health care in various countries are deeply convinced, can be corrected by rational combination of conventional and modern systems of medicine.

Ayurveda as a traditional medicine system, basing on scientific knowledge of individual peculiarities of human organism, interrelations thereof with environment, seasonal and weather effects thereon and correct nutrition shall allow real conduction of PREVENTION OF DISEASES and MAINTAINING the body in HEALTHY STATE.

Ayurveda’s predictive approach based on the constitutional knowledge about human being shall assist to solving a serious challenge of the modern life, namely birth of the full-rate vital generation, give opportunity to win non-infection diseases – cardiovascular, neurological, chronic respiratory and oncology diseases, diabetes and obesity. Preventive strengthening of immunity by ayurvedic means shall provide reducing of hazard of seasonal respiratory and allergic diseases.

Boost of priority of diseases prevention with use of ayurvedic methods shall allow reaching the highest health standards for people and overcoming obstacles on the way to well-being, improving health economy and social-economic development.

